# The Flaws and Future of Islet Volume Measurements

**DOI:** 10.1177/0963689718779898

**Published:** 2018-06-28

**Authors:** Han-Hung Huang, Stephen Harrington, Lisa Stehno-Bittel

**Affiliations:** 1 Angelo State University, Texas Tech University System, San Angelo, TX, USA; 2 Likarda, LLC, Kansas City, MO, USA; 3 University of Kansas Medical Center, Kansas City, KS, USA

**Keywords:** islet, islet equivalent, dithizone, ATP, oxygen consumption rate, kansas method

## Abstract

When working with isolated islet preparations, measuring the volume of tissue is not a trivial matter. Islets come in a large range of sizes and are often contaminated with exocrine tissue. Many factors complicate the procedure, and yet knowledge of the islet volume is essential for predicting the success of an islet transplant or comparing experimental groups in the laboratory. In 1990, Ricordi presented the islet equivalency (IEQ), defined as one IEQ equaling a single spherical islet of 150 μm in diameter. The method for estimating IEQ was developed by visualizing islets in a microscope, estimating their diameter in 50 μm categories and calculating a total volume for the preparation. Shortly after its introduction, the IEQ was adopted as the standard method for islet volume measurements. It has helped to advance research in the field by providing a useful tool improving the reproducibility of islet research and eventually the success of clinical islet transplants. However, the accuracy of the IEQ method has been questioned for years and many alternatives have been proposed, but none have been able to replace the widespread use of the IEQ. This article reviews the history of the IEQ, and discusses the benefits and failings of the measurement. A thorough evaluation of alternatives for estimating islet volume is provided along with the steps needed to uniformly move to an improved method of islet volume estimation. The lessons learned from islet researchers may serve as a guide for other fields of regenerative medicine as cell clusters become a more attractive therapeutic option.

## Islets and the Significance of Volume Measurements

Islets of Langerhans are small clusters of endocrine cells found in the pancreas, surrounded by exocrine tissue. They contain the only insulin-producing cells of the body (the β-cells), along with other hormone-releasing cells including glucagon-positive α-cells, somatostatin-positive δ-cells, ghrelin-positive ∊-cells and cells that produce pancreatic polypeptide. When islet function is sufficiently compromised, either by autoimmunity or other means, diabetes ensues. The first report in 1869 of cells within the pancreas that functioned beyond producing digestive enzymes came from the German medical student, Paul Langerhans. He found small round clusters of cells that stained differently than the rest of the pancreas, but whose function was unknown^1^. After 20 years, the physiologist, Oskar Minkowski and the physician Joseph von Mering showed that when the pancreas was removed from a dog, it became diabetic^2^.

Scientific advances rapidly expanded the understanding of the pancreatic endocrine function and its relationship to diabetes, ultimately leading to the purification of insulin from islets by Dr. Frederick Banting and medical student Charles Best along with biochemist James Collip^2^. Subsequently, the process of extracting bovine or porcine insulin became routine, producing large quantities, able to treat people with type 1 diabetes in North America. Yet, the isolation of intact islets remained a challenge. It was not until the 1960s and 70s that groups, including the laboratory of Dr. Paul Lacy, developed a procedure to isolate islets without massive cellular damage and transplant them into diabetic rats, reversing the diabetes^3,4^. At that point, the ability to measure and control the volume of islet tissue became essential.

Islets are naturally formed within the pancreas as spherical structures ranging in sizes from 30 μm to >400 μm in diameter^5–8^. This range of diameters appears relatively similar across species with the exception of rabbits, cats, and birds, which have smaller islets with maximum diameters of <200 μm. Table 1 summarizes findings from 24 studies reporting either the size range or the average diameter of islets from different species. To calculate the range of diameters from multiple sources, the maximum range across studies was recorded. When results from specific studies were dramatically different from a group, they were listed in Table 1 separately. Feline islets have been reported to be difficult to isolate and smaller in size, but publications of their average diameters are missing. Porcine islets were initially reported as small, but the results were likely due to suboptimal enzymatic isolation procedures and small samples sizes^9^, since publications using different techniques have obtained larger porcine islets in the range of 50–250 μm^10–12^. For human islets, research laboratories have reported islet diameters of <200 μm^8,9^. However, in one case the researchers used only one donor for their analysis^9^. Our own work surveying islets from 17 human donors (11 male and 8 female) found a range of islet diameters of 50–350 μm^13^, in agreement with later work comprised of over 200 human donor pancreata with average diameters close to 100 μm^14,15^. Other studies have identified even larger human islets, confirming sizes greater than 400 μm in diameter, but never providing an upper value for the range^5,16^. It is important to note that all of the values listed in Table 1 are from isolated islets, which may differ from islets within the pancreas. A thorough study of islet size in situ demonstrated only a slight decrease compared with diameters measured after isolation^15^. While it is typically assumed that the size range in situ is closer to the in vivo condition, the act of fixing and slicing tissues for in situ staining can also alter tissue morphology, so the exact in vivo islet size range is not certain.

**Table 1.** Average islet diameters along with size ranges, when available, are listed according to species.

Species	Diameter range (μm)	Average diameter (μm)	References
Human	30 to >400	108 ± 6	^5,6,8,13,15,16,17–19^
	20–180	50 ± 29	^9*^
Rat	30–350	115 ± 5	^20–22^
Mouse	20–350	116 ± 80	^9,23,24^
		154 ± 47	^25^^*^
Monkey	25–340	67 ± 38	^9,26^
Adult pig	50–250	156 ± 8	^8,11,12,27,28^
	20–90	49 ± 15	^9*^
Fetal pig	NR	83 ± 1	^10^
Rabbit	25-160	64 ± 28	^9^
Canine	50–375	158 ± 2	^29^
Goat	NR	50 ± 250	^30^
Bird	10–50	24 ± 6	^9^

For the size range, the maximum range across studies was recorded.

NR: not reported.

* When results from specific studies were dramatically different from others in that species, that report was listed separately.

Owing to inherent variations in size within and across species, an accurate method to estimate the volume of isolated islets in a preparation is essential. One cannot compare one laboratory experiment with another, or examine the effects of an experimental procedure with respect to a control group, without knowledge of the islet volume used in each condition. In the clinical setting, knowledge of the total islet volume transplanted is essential, and often predicts the success of the transplant procedure^31,32^. In fact, in single-donor transplants, the insulin requirements of the recipient at the time of the transplant and the volume of islets transplanted were the two factors that correlated with successful transplantation and insulin independence^33^. Conversely, an excessive volume of islets transplanted can put the recipient at risk for elevated portal pressure and internal bleeding^34^, because currently islets are infused into the portal vein for most human islet transplants. Therefore, the correct dose (volume) of islets is essential for a successful transplant.

Not only is total islet volume important in the success of transplants, the size of those islets is vitally important. Our lab showed that small islets (<125 μm) resulted in more successful islet transplants compared with the same volume of large islets in rats^22^. That finding was subsequently corroborated in mice^24,35^, rats^21^, goats^30^, and humans^36–38^. The improved outcomes with smaller islets is presumed to be due to better diffusion characteristics, because core cell death can quickly be measured in isolated islets >150 mm in diameter^39^. Factors beyond core cell death may also play a role. We showed that small human islets contained statistically more insulin-containing β-cells than large islets^40^, and other labs have documented smaller islet cells in the human pancreas that could also alter β-cell density between different sizes of islets^41^. While small human islets contribute minimally to the total islet volume, when calculated by islet equivalency (IEQ), the same is not true for other species. For example, our own experience working with canine islets, determined that islets <50 μm in diameter make up 16% of the total islet preparation.

## History of the IEQ

In the 1980s, a method for identifying islet cells, by staining with dithizone, provided an avenue for differentiating endocrine from exocrine tissue in a preparation^42^. While this technique helped determine the purity of an islet prep, there was little agreement on a standard method for quantifying islet mass. In 1990, Ricordi, along with a distinguished list of contributors, proposed the islet equivalent (IEQ) at the Second Congress of the International Pancreas and Islet Transplantation Association, as a means of normalizing islet volume^43^. This procedure standardized islet volume measurements and greatly enhanced islet research. It is based on the calculation that one IEQ corresponds to the tissue volume of a perfectly spherical islet with a diameter of 150 µm^43^. In 2010, an extensive study by Bonner-Weir’s lab estimated that one IEQ was comprised of 1560 individual cells^44^.

**Fig. 1.**
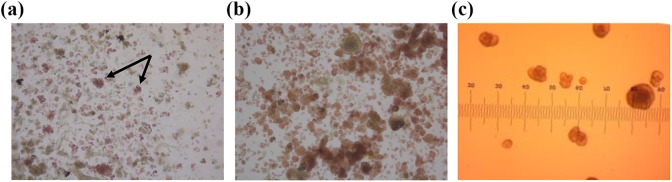
Examples of isolated islet preparations. (a) An example of a crude feline islet preparation stained with dithizone (red hue) to differentiate endocrine tissue (islets) from exocrine tissue. The significant variation in size and shape of the red-stained islets can be clearly seen in the image, along with variation in the intensity of the dithizone stain. (b) An example of a canine islet prep stained with dithizone. By comparison the islets are larger in size, but also non-uniformly shaped or stained. (c) Dithizone-stained human islets viewed through a microscope binocular with a grid used to categorize islet sizes into bins for IEQ calculations. IEQ: islet equivalency.

The procedure outlined by Ricordi is relatively easy to use and requires little in the way of instruments. First, a sample of the dithizone-stained islets are viewed under a brightfield microscope^45^, allowing the researcher to differentiate the islets (stained red) from the exocrine cells. Fig. 1(a) provides an example of a feline islet isolation prep with a mixture of islets (stained red) and exocrine tissue (brown). Feline islets tend to be small and do not contain a smooth spherical perimeter. Arrows point to a few of the larger feline islets. It is important to note that there is also variation in the intensity of the dithizone staining, with some islets appearing deep red, and others containing a pink hue. In general, canine islets (Fig. 1(b)) are larger, but also illustrate few truly spherical shapes. Individual islets are counted and their diameters estimated using the size grid in the microscope binocular. Fig. 1(c) shows human islets within the microscope’s binocular grid, which is the method used by technicians to place them in size categories. With a 4× objective, the divisions on the eyepiece are calibrated to 50 μm. Rather than recording exact diameters for each islet, the technician simply “bins” the islets into 50 μm increments from 50 to 350 μm diameters. The number of islets in each size bin is multiplied by a unique factor that converts the islet number and diameter into IEQs^43,46^. The result is a simple method that can be completed in a timely manner.

After its introduction, the Ricordi method rapidly became the standard volume estimation procedure and remained that way through the present. Currently, the IEQ is used to estimate the yield of islets isolated from a donor, and the IEQ per kg of body weight is the unit used to report the graft amount transplanted into the patient^4,31,47–52^. In the research laboratory, IEQ is commonly applied to normalize the volume of islet tissue between preparations for functional assays such as insulin secretion^9,22,37,53–56^.

## Issues with IEQ Counts

In 2010 a multicenter study designed to examine the IEQ procedure in detail was published^57^. The same micrographs of human islets were scored for IEQ by 36 different technicians at 8 clinical sites. Overestimation of the IEQ occurred approximately 50% of the time, and the intra-technician coefficients of variation from one repeat count ranged from 0 (technician placed the same islet into the same size category on multiple occasions) to a maximum of approximately 43%^57^. The wide variation is understandable, given the subjective nature of the binning process illustrated in Fig. 1(c). The results from the multicenter study illuminated the difficulty in determining islet volume using the method, and underscored the poor validity and reliability of IEQ measurements.

From its introduction to the present, the accuracy of the IEQ measurement has been challenged^5,7,20,57–59^. One of the most consistent criticisms of the IEQ has been its mathematical basis on the ideally spherical 150 μm diameter islet equaling one IEQ. In fact, most islets are not spherical, but are irregularly shaped, both in situ and in culture^20,49,59–61^. Fig. 1 illustrates that the spherical nature of islets can vary between species, and also between preparations.

Our own work confirmed the irregularity of human islets using video capture as they were rolled through a custom-made chamber^13^. A measurement of the three largest dimensions in mutually perpendicular directions of the isolated islets was calculated. In a perfect sphere, the three major dimensions *a*, *b*, and *c* would be equal (*a* = *b* = *c*). However, the measured ratios were an average of *b*/*a* = 0.82 and *c*/*a* = 0.7, suggesting that islets, especially large islets, are predominantly ellipsoidal in shape^13^. The findings support independent research published in dissertation form, showing diameter ratio values of an average of 0.6^60^.

Islet circularity is another method of estimating the overall spherical shape of islets. Circularity varies depending on the overall size of the islet with large islets having less circularity^18,20^. Our own unpublished calculations of circularity, based on two-dimensional microscopic images, indicated that small islets had a circularity value of 0.801 ± 0.006, while large islets isolated from the same rats had an average circularity of 0.740 ± 0.029. There have been reports that the location within the pancreas and the presence or absence of disease can also affect islet circularity^18^.

An additional problem with the IEQ measurements is the process of binning the islets into 50 μm size categories, rather than using the actual diameter measurement. In fact, the procedure of binning islets into 50 μm size ranges may alone lead to an overestimation of IEQ^5,59^, meaning that the technicians estimate the diameters to be larger than they are, and place them in larger diameter categories during binning. To test this hypothesis, we measured the diameter of individual islets, and then grouped them following the Ricordi size categories. The islets were subsequently dispersed into single cells and individual cell numbers were counted using an automated cell counter. The cell number was divided by the original islet diameter or by the Ricordi diameter category assigned by the technician. When the cells/islet diameter were calculated, the average was 3.5 ± 0.3 cells/μm. However, when the same data were normalized to the Ricordi islet diameter category the value was 2.8 ± 0.2 cells/μm (*n* = 4 rats). Although not statistically different, the results demonstrate that normalizing the data by 50 μm islets size groupings, rather than the actual diameter, caused a 20% under-estimation of cell density in our laboratory. Further evaluation of our own internal process determined that the staff were overestimating the actual diameter during the binning process, thus skewing the data.

In addition, the original Ricordi binning procedure excluded islets below 50 μm in diameter. This limitation was understandable, because in humans and rodents the small islets represent a minimal percentage of the total volume. In rodents more than 50% of the total β-cell area comes from the largest 2% of islets^62^. Yet, that relationship is not true for all species. As shown in Table 1, the average islet diameter in certain animals such as birds, rabbits, monkeys, and pigs is lower, shifting the size distribution towards smaller islets. Further, small islets appear to be the most plastic, able to respond to conditions such as pregnancy and aging, and are spared in type 2 diabetes^63^, but affected during disease states such as type 1 diabetes^64^. Thus, excluding them from the volume calculation might underestimate the importance of those islets.

In 2009, Ricordi’s laboratory recognized that ignoring a large percentage of islets could affect the IEQ conversion. Adjustments were made to the original IEQ calculations, along with the addition of a conversion factor for islets under 50 μm, resulting in a downward adjustment of the IEQ values^5^. However, when theoretical cell numbers were plotted for islets of varying diameters using the original method and the revised calculations, the actual Buchwald correction was small and significant differences could only be detected for large islets (over 250 μm in diameter)^13,20^. A year later another mathematical adjustment to the conversion equation was proposed by Kin^65^. Again, the changes resulted in minor adjustments to the overall IEQ counts.

## Attempts at Improving Islet Volume Measurements

To avoid the subjective nature of diameter determination and size binning that is inherent in the IEQ procedure, several digital image analysis methods have been proposed to replace the manual procedure^23,49,57,61,66–68^. These procedures aim to improve the reliability of the islet count and reduce the time required to estimate islet volume for transplantation. Islet volumes calculated from images using MetaMorph and ImageJ software have been shown to be reliable, yet the final step in the described process still bins the islets into size categories and converts them to IEQ values. In an important paper comparing manual size estimation with computer-assisted digital image analysis, the variability of standard manual counting methods was clear. The digital process was superior for determining purity and viability in a good manufacturing practice (GMP)-compliant manner^69^.

While the need for automation, along with digital imaging was identified as early as 1995^70^, a fully automated instrument for islet volume assessment was not developed until 2016^14^. The Islet Cell Counter is an integrated imaging device with software created specifically to analyze the dithizone-stained islets, providing both IEQ and purity values for each sample^14^. The instrument is GMP compliant and has been shown to be superior to manual counting for islets between 50 and 400 μm in diameter. However, the software bins the islet diameters into the same 50 μm groups and calculates an IEQ as the final output. Other groups with unique digital analysis procedures also incorporate a final step in volume estimation with a conversion to IEQ^14,23,68,69,71^. As stated earlier the process of binning islets into categories based on their diameter may introduce its own errors. Analysis of islet images based on individual islet diameters or islet area illustrated that when using area instead of diameter, the total IEQ changed^14^. One group from Prague used digital imaging with conversion based on an assumed ellipsoidal shape, rather than the elusive spherical islet^61^. More recently another lab has confirmed that they are working on new algorithms to better account for the non-spherical shape of islets^71^.

## Non-IEQ Methods

Methods for determination of islet volume that avoid the IEQ altogether have been developed, but none have been widely adapted. In 1992, a measurement of total zinc content was proposed as a way to eliminate the visual estimation of islet diameters^72^. A fluorescent zinc tag can be used to quickly stain for intracellular zinc levels. One concern was that, while zinc is in higher concentrations in islet cells, it is present in all cells, including exocrine tissue. However, experiments purposefully contaminating samples with up to 50% exocrine tissue, did not appreciably alter the results. The procedure was relatively simple, required only a common plate reader, and worked well in rat and human islet isolations^72^. Yet it was never widely accepted as a popular method for volume normalization or purity assessment.

Counting nuclei with stains like Hoechst or 4’,6-diamidino-2-phenylindole (DAPI) or using automation to count liberated nuclei have all been proposed as methods for determining islet cell numbers^7,73^. Alternatively, DNA content can be measured as another indicator of total cells in the sample. The validation of the nuclei counts obtained through flow cytometry has been compared with DNA concentration measurements and found to be linear. The advantage with both DNA content measurements and nuclei counting is that they are less subjective or prone to error^73,74^. However, neither differentiate between islet and non-islet cells. Thus, additional tests for sample purity must be completed^44^. When nuclei counting was added to microscopic evaluation of islet purity, the precision was high and when compared with manual IEQ calculations, the results once again showed that IEQ measurements over-estimate the islet volume by up to 90%^44^.

Unfortunately, DNA measurements have their own challenges. Colton et al. conducted a thorough comparison of DNA measurements using different fluorescent probes and found significant differences between assays and the sources of the islets, which might be due to the DNA degradation in shipped islets^73^. We have used total DNA measurements to evaluate the accuracy of the Ricordi IEQ method. Our results show wide variations in the DNA/IEQ, depending on the size of the islets and the quality of the preparation^20^, but that variation was resolved when the same DNA data were normalized to cell number.

Adenosine triphosphate (ATP), which is essential in insulin secretion, is an alternative indicator of islet volume, and can be measured directly with bioluminescent assays. ATP and adenosine diphosphate (ADP) measurements have been shown to closely correlate with insulin secretion, but not the release of glucagon^75^. Results from several labs have suggested that the ATP/ADP ratio is a strong predictor for the success of islet transplants and should be central to the islet quality control prior to transplantation^76–80^. Others have suggested that the ratio of ATP/DNA is more predictive of transplant outcomes than ATP/ADP^81^. Like DNA and zinc, one might assume that measurements of ATP and ADP are not specific to endocrine tissue. However, islet ATP and ADP are somewhat different, because within the islet the ATP/ADP ratio is responsive to stimuli, which differentiates it from other cells^76^. Thus, a high ATP/ADP ratio typically indicates healthy islet cells and can be associated with better insulin secretion^75^. In most settings, ATP and ADP would be used as an estimate of islet health and function, not volume. However, we should not eliminate it as a measure of islet volume, if an accurate calibration can be made between these values and current islet volume measurements.

Another metabolic measure is the oxygen consumption rate (OCR) first applied to islets in the late 1990s^82,83^. A special OCR chamber, equipped with fiber-optic sensors that measure oxygen partial pressure over time, is required for the procedure. When tested prior to transplants into diabetic mice, the OCR output divided by DNA content was 89% sensitive and 77% specific in predicting the reversal of diabetes after the islet transplant^82^. The sample of islets needed to reliably use OCR as a predictive tool is relatively large (500–2000 IEQ). While this may be only a small fraction of islets used in a clinical islet transplant, it would be a large percentage of islets used in a rodent study, for example. Thus, its utility is likely applicable only to the clinical setting, and again may be best suited to analyze the functional islet mass. In fact, both ATP measurements and OCR may be better measures of the volume of functional islets. Studies have shown that in vivo β-cell function and mass can change independently. In type 1 diabetes, function is lost first, while islet volume loss occurs later in the disease progression (reviewed by Chen et al.^84^). Thus, functional islet volume may be a more important parameter to measure when trying to predict transplant success.

Large particle flow cytometry offers another option of islet volume measurements. These instruments are based on flow cytometry principles, but can analyze and sort particles ranging from 0.4 to 1.5 mm in diameter. They can count and sort islets based on size or fluorescent markers, and they are available from several manufacturers. We utilized the technology to separate large and small islets based on a 100 μm diameter cut-off point^39^. Sorting the islets through the large particle cytometer did not alter function or viability when compared with manual separation of the same samples. The instrument provides data on the Time of Flight (a measure of islet diameter), extinction (indicating individual islet density) and can also quantify fluorescence tags^39^. Fernandez et al. ^85^ utilized large particle flow cytometry to make islet volume measurements. Unfortunately, rather than converting Time of Flight directly into islet diameters and then into volume, the authors converted the Time of Flight values back to IEQs.

As explained previously, categorizing islet diameter in 50 μm groups offers an inferior method to estimate islet volume. We developed a new volume estimation procedure based on cell numbers that avoided the IEQ calculations^13,20^. Using rat islets, we placed nearly 350 individual islets, ranging in size from 50 to 350 μm in diameter, into wells with a single islet per well. After measuring two to four diameters for each islet, we dissociated them into single cells and counted the cells per well using an automated imaging system^20^. All of this was done within the same well and without washes to avoid loss of cells. A calibration curve was then created, based not on theoretical islet shapes, but on measured cell numbers. The equation allows cell numbers to be calculated from islet diameters of any specific size, rather than requiring the binning step. The user measures the islet diameter and plugs it into a conversion equation that calculates the number of cells in that islet. We call this method of volume estimation the Kansas method.

The procedure requires no special equipment and can be used with any digital imaging system or manual measurements. Furthermore, it no longer requires binning of islet diameters into 50 μm categories, but can convert an islet of any size directly into an estimated cell number. We determined that separate cell number conversion equations were necessary for human^13^ and rat islets^20^. The Kansas method has been integrated into spreadsheets that automatically calculate cell numbers from any measured islet diameter between 20 and 350 μm and has been placed on our website for free download at http://www.ptrs.kumc.edu/kansasmethod/. Different spreadsheets are available for human and rat conversions. To reduce errors further, the approach can blend with automated imaging for exact diameter measurements fed into the Kansas method spreadsheet.

In previous publications, we illustrated the overestimation of the volume of large islets with IEQ and demonstrated that using the Kansas method corrected the error. More recently, we have found that the Kansas method predicted successful islet xenotransplants in a diabetic mouse model, while converting the islet dose based on IEQ failed. Table 2 summarizes a series of transplants of canine islets into diabetic NOD-SCID mice in which the transplanted volume was calculated as cells/mouse or IEQ/mouse. When calculated as IEQ, mice from each group receiving a low dose (2500 IEQ), a moderate dose (3500 IEQ) and a high dose (4000 IEQ) were reversed of diabetes. Thus dose, when calculated by IEQ did not correlate to transplant outcome. However, when the same transplants were calculated based on cell numbers, the values provided a data-driven cut-off point for successful transplants. No transplants under 5.13 M fully reversed diabetes, while transplants with over 6 million cells were successful 100% of the time. We are expanding this study with more transplants in the 5.5–6.25 M cell range to refine the correlation between cell number and transplant success in the rodent model. One explanation for the difference between the results with normalization by IEQ or cell number is that the islet preps containing a higher percentage of small islets were more likely to be successful^36^. In fact transplant Group 1 contained 57% of the islets under 100 μm in diameter, while Group 3 contained 73% small islets.

**Table 2.** Results from studies transplanting canine islets into diabetic NOD-SCID mice.

Transplant group	Outcome	Cells/mouse	IEQ/mouse
1	100% partial response	5.00 M	2500
2	33% failure	5.13 M	3500
	33% partial response		
	33% normal glycemia		
3	100% normal glycemia	6.17 M	2500
4	100% normal glycemia	7.25 M	4000

Transplants on the left side of the table show the volume of islets transplanted into the mice based on IEQ. When calculated as IEQ, some of the mice in each group responded fully to the transplant with normal glycemia. However, when the same transplants were calculated based on cell numbers (right side of table), the results suggest that transplants with 6.17 million cells or more were successful 100% of the time. *N* = 10 mice.

IEQ: islet equivalency.

Calculating islet volumes based on cell numbers rather than IEQ results in several shifts in thinking about islet preps. For example, IEQ values for human islet preparations have consistently shown that small islets make up a very minimal percentage of the total islet volume. This thought is so pervasive, that it is rarely questioned. When we analyzed human islet preps using IEQ, we found that those islets under 50 μm in diameter made up only 6.6% of the total volume. However, when the same preparations are first converted to cell number, the contribution of small islets (≤50 μm) was 17% of the total. Thus, utilizing the cell number conversions in the Kansas method may help to clarify some of the contradictory data found in the literature.

## Sampling Error

To suggest that abandoning the IEQ volume measurement for one of the newer alternatives will correct all errors inherent in the volume estimation process would be wrong. While the non-IEQ methods of volume estimation can overcome some of the flaws of the IEQ method, all attempts to determine islet volume face the issue of sampling error. Because the islets are heavy and quickly settle in liquid, the time, location, and even speed of pipetting will alter the number of islets in the aliquot and thus introduce errors in volume estimations. Current studies designed to measure inter-rater reliability between technicians conducting islet testing, typically use images of islet preps to test the technicians, completely missing the additional error introduced by different pipetting techniques. While we, and others, have tried to minimize the error with strict standard operating procedures and multiple samples per prep, there appears to be no way to avoid sampling error currently. Further, large dilution factors used in the clinical setting only exaggerate the issue.

## Conclusion

Over the past 25 years, the IEQ measurement to estimate islet volume has been a useful tool that has moved the fields of islet research and clinical islet transplants forward. Yet today there are likely better options, including computer programs for automated area or diameter, or fluorescence/luminescence measurements that remove the human error intrinsic in the IEQ procedure. Islet transplants, and all of the supporting research, have been at the forefront of the cell therapy field. As other cell therapies find their way to clinical reality, it is likely that several of them will also be based on cell clusters. Thus, accurate methods to estimate transplant volumes of islets could have implications for various regenerative medicine therapies. Unfortunately, improved procedures will never be widely adopted until leaders in the islet transplant field advocate advanced methods and pick one of the many scientifically sound options as the new gold standard. Until then, we are all left with a less optimal procedure that we cannot avoid if we want to disseminate our laboratory studies or conduct clinical trials.
